# Integrative Genome-Wide Association Study (GWAS), Transcriptome, and Sequence Variation Analyses Reveal Candidate Genes Controlling Barley Grain Length

**DOI:** 10.3390/genes17060615

**Published:** 2026-05-29

**Authors:** Panpan Li, Zhiguo Xiang, Dan Zhang, Xianlin Zhao, Zhidan Zuo, Hongshan Yang, Dongyang Liu, Yongying Zhao

**Affiliations:** Wheat Institute, Henan Academy of Agricultural Sciences (HAAS), Zhengzhou 450002, China; li_pp1203@163.com (P.L.);

**Keywords:** barley, grain length, GWAS, RNA-seq, candidate genes

## Abstract

Background: Grain length is a key determinant of yield and quality in barley (*Hordeum vulgare* L.) and is typically governed by complex quantitative traits. Methods: In this study, a diverse natural population comprising 198 barley accessions was evaluated across two years to investigate the genetic basis of grain length. Results: Phenotypic analysis revealed continuous variation with near-normal distribution, indicating polygenic control. Genome-wide association study (GWAS) identified 84 stable single nucleotide polymorphism (SNP) loci significantly associated with grain length, predominantly enriched on chromosome 7. RNA sequencing (RNA-seq) was conducted using two contrasting genotypes at four developmental stages. Differentially expressed genes (DEGs) were mainly enriched in structural constituent of chromatin, protein heterodimerization activity, and the starch and sucrose metabolism. Integration of GWAS and RNA-seq identified 7 key candidate genes seven key candidate genes, including *LOC123412467*, *LOC123408579*, *LOC123407599*, *LOC123410619*, *LOC123410954*, *LOC123411868*, and *LOC123426274*. Sequence variation analysis further revealed functional polymorphisms, including non-synonymous mutations. The sequencing results show that *LOC123412467* and *LOC123410619* exhibited consistent allelic variation between long-grain and short-grain accessions, while *LOC123426274* displayed stable differential expression across developmental stages, indicating their potential roles as key genes controlling grain length. Conclusions: Collectively, these findings suggest that chromosome 7 contains major regulatory loci controlling barley grain length and demonstrate that integrative multi-omics analysis is an effective strategy for identifying high-confidence candidate genes associated with complex agronomic traits. This study provides valuable insights into the genetic basis of grain length and offers key candidate genes for barley molecular breeding.

## 1. Introduction

Barley (*Hordeum vulgare* L.) is a diploid, annual, predominantly self-pollinating cereal crop belonging to the family Gramineae Poaceae, tribe Triticeae, and genus *Hordeum*. It is recognized as one of the earliest domesticated crops, having been derived from its wild progenitor (*Hordeum spontaneum*), and ranks as the fourth most widely cultivated cereal worldwide after wheat, maize, and rice. Barley is extensively grown across diverse agro-ecological regions, including the European Union, Russia, Canada, Australia, and Ukraine, where it plays a crucial role in food, feed, and malting industries. In China, barley has a cultivation history exceeding 5000 years and has undergone long-term artificial selection, resulting in considerable improvement of agronomic traits. However, its genetic diversity remains relatively limited, with underutilization of favorable alleles, thereby constraining further genetic gain in yield and quality improvement [1,2]. Grain yield in barley is a complex trait determined by multiple components, among which grain size—particularly grain length—is a key determinant affecting both yield potential and end-use quality [3]. Grain length contributes directly to thousand-kernel weight and is closely associated with grain morphology and processing characteristics, making it an important target in modern barley breeding programs [4,5].

As a typical quantitative trait, grain length is controlled by multiple loci with small-to-moderate effects and is strongly influenced by environmental factors and genotype–environment interactions [6,7]. Genome-wide association studies (GWAS) have become an effective approach for dissecting the genetic architecture of complex traits by leveraging natural variation and historical recombination [8,9,10]. Compared with traditional linkage mapping, GWAS provides higher resolution and captures broader allelic diversity, enabling the identification of loci associated with agronomic traits in diverse germplasm collections.

In barley, previous GWAS have identified multiple genomic regions associated with grain size and yield-related traits [11,12,13]. Advances in high-density genotyping platforms and high-quality reference genome assemblies have further improved mapping precision [1,14]. However, GWAS signals alone are often insufficient to resolve causal genes due to extensive linkage disequilibrium (LD), which may span large genomic intervals containing multiple candidate genes [15]. Therefore, integrating additional layers of biological evidence is essential to refine candidate gene identification.

Transcriptome profiling using RNA sequencing (RNA-seq) provides complementary insights into gene expression dynamics and regulatory networks during plant development [16,17]. In cereal crops, RNA-seq studies have revealed that grain development involves coordinated regulation of pathways related to cell division, cell expansion, hormone signaling, and carbohydrate metabolism [18,19]. Importantly, the integrative analysis of GWAS and RNA-seq facilitates the identification of genes jointly supported by genetic association signals and transcriptional dynamics, thereby improving the accuracy and confidence of candidate gene prioritization [20,21,22]. A recent study in barley also successfully identified grain size-related candidate genes through combined GWAS and transcriptome analysis, further demonstrating the effectiveness of integrative multi-omics approaches for complex trait dissection [23].

Beyond transcriptional evidence, protein functional annotation and sequence homology analyses provide critical insights into gene function. In rice and wheat, numerous grain size-related genes have been identified, including *GS3*, *GS2/GL2*, *GS5*, and *GL7/GW7*, which regulate grain morphology through diverse mechanisms such as ubiquitin-mediated protein degradation, transcriptional regulation, and hormone signaling pathways [24,25,26,27]. The conservation of functional domains across species suggests that homologous genes in barley may play similar roles in regulating grain development.

In addition, sequence variation analysis, particularly the identification of single nucleotide polymorphisms (SNPs) and insertion–deletion polymorphisms (InDels), provides direct evidence linking genetic variation to phenotypic diversity [28,29]. Non-synonymous mutations may alter protein structure and function, whereas regulatory variants can influence gene expression patterns, highlighting the importance of integrating sequence-level variation with functional data.

Despite these advances, the genetic and molecular mechanisms underlying grain length variation in barley remain incompletely understood. Most previous studies have relied on single-omics approaches, which may lead to high false-positive rates or overlook key regulatory components [30]. Moreover, the relationships among genetic variation, transcriptional regulation, and protein function have not been systematically integrated, limiting a comprehensive understanding of the regulatory networks controlling grain development.

To address these limitations, the present study employed an integrative multi-omics strategy to dissect the genetic basis of grain length in barley. A diverse natural population comprising 198 accessions was phenotyped across multiple environments and subjected to GWAS to identify genomic regions associated with grain length. RNA-seq analysis of extreme phenotypes at multiple developmental stages was conducted to characterize transcriptional dynamics and identify differentially expressed genes. Protein sequence homology analysis was further performed to detect conserved functional domains among candidate genes, and targeted resequencing was used to identify sequence variants potentially underlying phenotypic differences.

By integrating genetic association signals, transcriptional profiles, protein structural information, and sequence variation data, this study aimed to (i) elucidate the genetic architecture of grain length; (ii) characterize stage-specific transcriptional dynamics during grain development; (iii) identify key regulatory pathways and transcription factors; and (iv) prioritize high-confidence key candidate genes through multi-layered evidence integration. The findings will provide new insights into the molecular mechanisms governing grain length and highlight potential targets for molecular breeding, while also offering a generalizable framework for dissecting complex traits in crop species.

## 2. Materials and Methods

### 2.1. Plant Materials and Phenotyping

A total of 198 barley (*H. vulgare*) accessions with diverse genetic backgrounds were grown in 2021 and 2022 at the Modern Agricultural Research and Development Base, Henan, China (113.704663° E, 35.005322° N). The experiment followed a randomized complete block design with three biological replicates under standard agronomic management. At maturity, grains from the central spikes were collected for phenotypic evaluation. Grain length and width were measured using an SC-G automatic seed analyzer (Wseen Testing Technology Co., Ltd., Hangzhou, China), and thousand-kernel weight (TKW) was determined using an electronic seed counter (Model PB203-E, METTLER TOLEDO Co., Ltd., Shanghai, China). Each trait was measured with three technical replicates and averaged. Phenotypic data across environments were integrated using a linear mixed model to calculate best linear unbiased predictions (BLUPs).

### 2.2. DNA Extraction and Sequencing

Based on grain length phenotypes, the 198 barley accessions were ranked, and 30 long-grain and 30 short-grain accessions (60 accessions in total) representing extreme phenotypes were selected for DNA extraction and sequencing. Leaf samples were collected at the grain filling stage (15–20 days after flowering), frozen in liquid nitrogen, and stored at −80 °C. Genomic DNA was extracted by Shanghai Personal Biotechnology Co., Ltd. (Shanghai, China). Using the CTAB method and assessed by agarose gel electrophoresis and a NanoDrop spectrophotometer (Thermo Fisher Scientific, Waltham, MA, USA). Approximately 1 μg of high-quality genomic DNA from each accession was used for dd-RAD library construction and sequencing. The dd-RAD libraries were constructed and sequenced on the Illumina NovaSeq platform (PE150) (Illumina Inc., San Diego, CA, USA). Raw reads were quality-checked using FastQC v0.20.0 and filtered using Trimmomatic v0.39 (sliding window: 5 bp; Q ≥ 20) to obtain high-quality clean reads.

### 2.3. SNP Calling and GWAS

Clean reads were aligned to the barley reference genome (Morex v3) using BWA-MEM v0.7.17, followed by sorting, indexing, and format conversion with SAMtools. Variant calling was performed using the GATKv4.2 pipeline, and raw variants were subjected to stringent quality control. High-confidence SNPs were retained by applying the following filtering criteria: minor allele frequency (MAF) ≥ 0.05 and missing rate ≤ 20%.

Genome-wide association analysis was conducted using a mixed linear model (MLM) implemented in TASSELv5.2 or the GAPITv3 package in Rv4.3.0, effectively controlling for both population structure and kinship. Population structure (Q matrix) was inferred via principal component analysis (PCA). A phylogenetic tree was constructed using the maximum likelihood method implemented in FastTreev2.1.11 (http://www.microbesonline.org/fasttree/) (accessed on 20 November 2025). Branch reliability was evaluated using bootstrap analysis with 1000 replications.

### 2.4. RNA Sequencing and Differential Expression Analysis

Two accessions with contrasting grain length phenotypes (WDM01925 and E720706) were selected. Grains were sampled at 7, 14, 21, and 28 days after flowering (DAF), with three biological replicates per stage. Total RNA was extracted using TRIzol (Invitrogen, Carlsbad, CA, USA) and assessed using NanoDrop (Thermo Fisher Scientific, Waltham, MA, USA) and Agilent 2100 Bioanalyzer (Shanghai Personal Biotechnology Co., Ltd., Shanghai, China). Libraries were sequenced on an Illumina platform (PE150) (Illumina Inc., San Diego, CA, USA). Clean reads were aligned to the reference genome (Morex v3) using HISAT2v2.2.1, and gene expression was quantified using featureCountsv2.0.1. Differential expression analysis was performed using DESeq2v1.42.0 with the model~genotype + developmental_stage.

### 2.5. Transcription Factor Analysis

DEGs were annotated against the Plant TFDB database to identify transcription factors and classify them into families. The distribution of TF families was summarized and visualized using Rv4.3.0.

### 2.6. Integrative Analysis of GWAS and RNA-Seq

Candidate genes were identified by integrating GWAS loci with DEGs. Genes located within significant GWAS regions and showing differential expression were prioritized. Protein sequences of known grain length-related genes (Table 1) were retrieved from NCBI, and candidate gene sequences were extracted using TBtools-IIv2.390 (Chen et al., Guangzhou, China). Homology analysis was performed to identify conserved domains and prioritize candidate genes.

### 2.7. Functional Enrichment Analysis

GO and KEGG enrichment analyses were performed using clusterProfilerv4.8.0.

### 2.8. Candidate Gene Validation

Based on grain length phenotypes, 30 long-grain and 30 short-grain accessions were selected. Candidate genes were amplified using gene-specific primers listed in Appendix A (Table A1) and sequenced by Tsingke Biotechnology Co., Ltd. (Beijing, China). Sequence alignment was performed using MegAlign (DNASTAR Lasergene v7.1, DNASTAR Inc., Madison, WI, USA) to identify polymorphisms.

### 2.9. Statistical Analyses

Phenotypic data were analyzed using Microsoft Excel Mondo 2016. Descriptive statistics, including mean, standard deviation, coefficient of variation, and frequency distribution, were calculated for grain length-related traits across different environments. One-way analysis of variance (ANOVA) followed by Duncan’s multiple range test (*p* ≤ 0.05) was performed to evaluate phenotypic variation among barley accessions. Best linear unbiased predictions (BLUPs) were calculated using a linear mixed model to minimize environmental effects. Broad-sense heritability was estimated based on variance components across environments.

For GWAS analysis, significance thresholds were determined using Bonferroni correction or −log10(*p*) values. Principal component analysis (PCA) was used to evaluate population structure and sample clustering. In RNA-seq analysis, differential expression analysis was performed using DESeq2, and genes with |log2 fold change| ≥ 1 and false discovery rate (FDR) < 0.05 were considered differentially expressed genes (DEGs). GO and KEGG enrichment analyses were conducted using the hypergeometric test with Benjamini–Hochberg correction, and terms with FDR < 0.05 were regarded as significantly enriched. Unless otherwise stated, statistical significance was defined at *p* < 0.05.

## 3. Results

### 3.1. Phenotypic Variation and Genetic Characteristics of Grain Length

**Figure 1:**
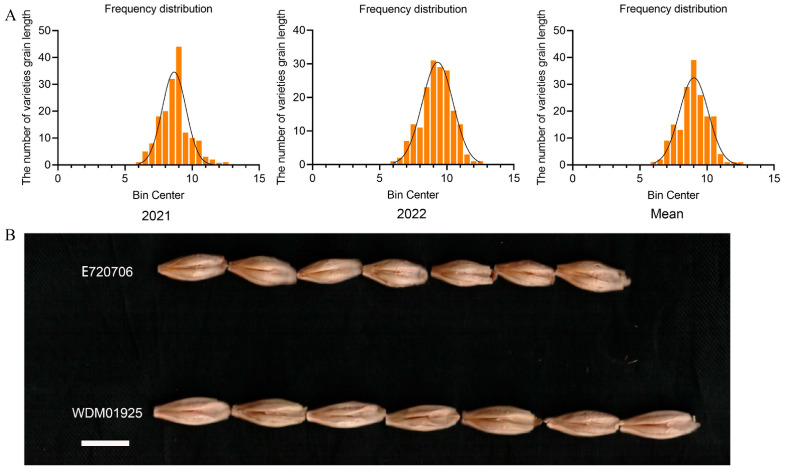
Distribution of grain length and representative grain phenotypes in the barley population. (**A**) Frequency distribution of grain length; (**B**) phenotypic variation. Bar = 0.5 cm.

A total of 198 barley accessions with diverse genetic backgrounds were evaluated for grain length in 2021 and 2022. The results show that grain length exhibited continuous variation across the population, which is consistent with the characteristics of a typical quantitative trait (Figure 1A). Representative grain phenotypes of selected accessions are shown in Figure 1B. Descriptive statistical analysis (Table 2) indicated that the mean grain length remained relatively stable across the two years, while the coefficients of variation exceeded 12%, suggesting that the population possessed substantial genetic diversity. The frequency distribution analysis further showed that grain length approximately followed a normal distribution, with skewness and kurtosis values close to zero, indicating that the trait may be controlled by multiple minor-effect genes.

Overall, these results indicate that the population exhibited appropriate phenotypic variation and genetic stability, and is therefore suitable for genome-wide association studies (GWAS).

### 3.2. Identification of GWAS Loci

#### 3.2.1. Population Structure and SNP Characteristics

**Figure 2:**
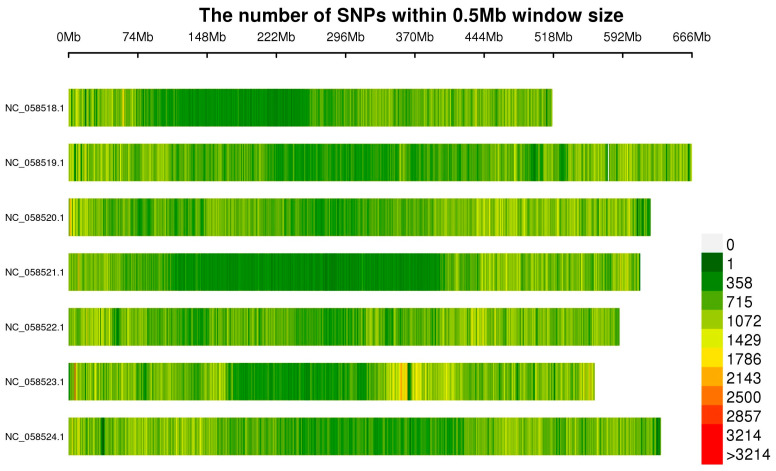
Distribution and density of SNPs across chromosomes. Polymorphism. The SNP density distribution plot illustrates the number of single nucleotide polymorphisms (SNPs) within 1 Mb sliding windows across the barley genome. NC_058518.1 to NC_058524.1 correspond to barley chromosomes 1H–7H, respectively.

Based on high-density genome-wide SNP markers, the population structure was systematically analyzed. After stringent quality control, a large number of high-quality SNPs were obtained and found to be evenly distributed across the seven chromosomes (Figure 2). The minor allele frequency (MAF) distribution was reasonable, indicating a high level of genetic diversity within the population.

**Figure 3:**
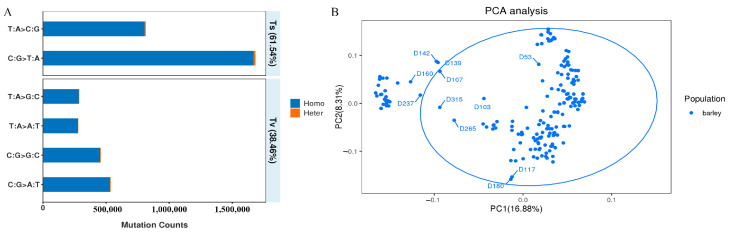
SNP mutation spectrum and principal component analysis (PCA). (**A**) Analysis of mutation types; (**B**) PCA analysis.

The distribution pattern of SNPs showed enrichment in chromosome arms and depletion in centromeric regions (Figure 2), which is consistent with recombination rate patterns. Analysis of mutation types (Figure 3A) showed that transitions (Ts) occurred more frequently than transversions (Tv), with a Ts/Tv ratio greater than 1, which is consistent with the general mutation characteristics of plant genomes.

**Figure 4:**
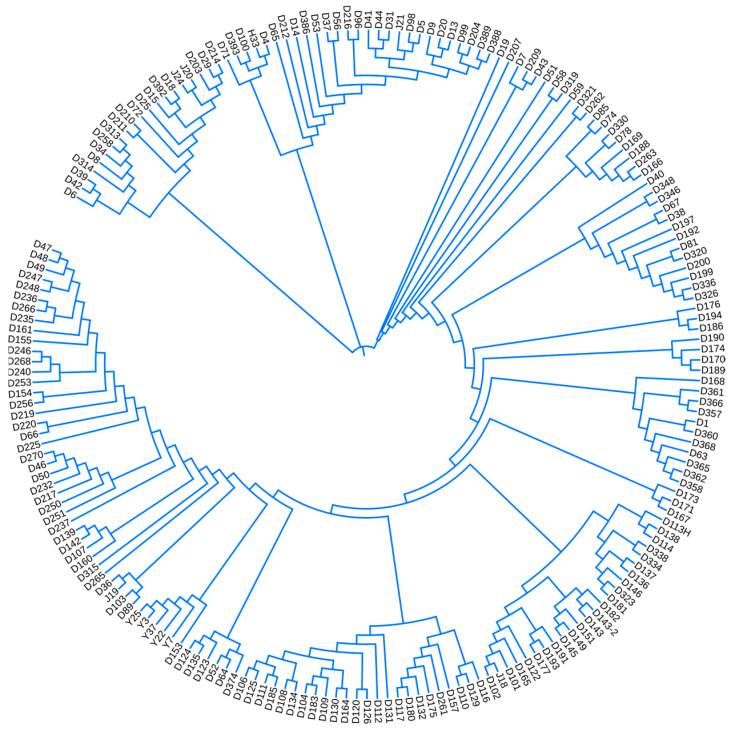
SNP-based phylogenetic tree analysis. Each branch represents an individual accession, and the branch length indicates the evolutionary distance between accessions, reflecting their degree of genetic divergence.

Principal component analysis (PCA) results show that the first three principal components explained 30.31% of the total genetic variation (Figure 3B), suggesting the presence of moderate population structure without clear stratification. Phylogenetic analysis showed results consistent with PCA (Figure 4), indicating clear genetic relationships among accessions. Such population characteristics minimize the confounding effects of structure and kinship in GWAS, thereby improving the accuracy of detecting loci associated with complex traits such as grain length. This provides a reliable genetic basis for the identification of significant SNPs and candidate intervals in subsequent analyses.

#### 3.2.2. Significant Loci and Candidate Interval Definition

**Figure 5:**
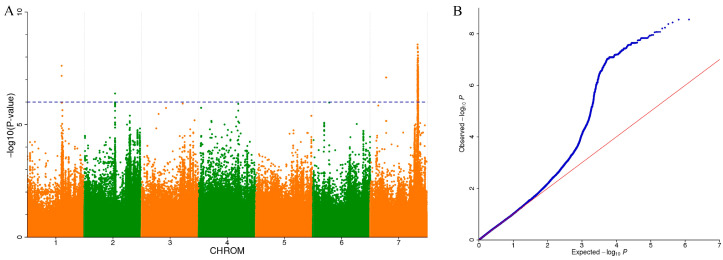
Manhattan and QQ plots of GWAS for grain length. (**A**) Manhattan plots of barley grain length; (**B**) QQ plots barley grain length. The dashed horizontal line represents the genome-wide significance threshold (*p* = 5 × 10^−8^), and alternating colors indicate different chromosomes.

The GWAS results show that multiple genomic regions were significantly associated with grain length (Figure 5A), indicating that grain length is a typical polygenic trait. Among these, the strongest and most significant association signals were detected on chromosome 7, suggesting that this region may harbor major-effect loci.

The quantile–quantile (QQ) plot (Figure 5B) showed that the observed values were generally consistent with the expected values, with deviation only in the tail region, indicating that the model effectively corrected for population structure and kinship effects, and that the results were reliable.

Under multi-environment conditions, a total of 84 SNP loci significantly associated with grain length were identified (Table 3). These loci were mainly distributed on chromosomes 1, 2, and 7, with a clear enrichment on chromosome 7.

**Figure 6:**
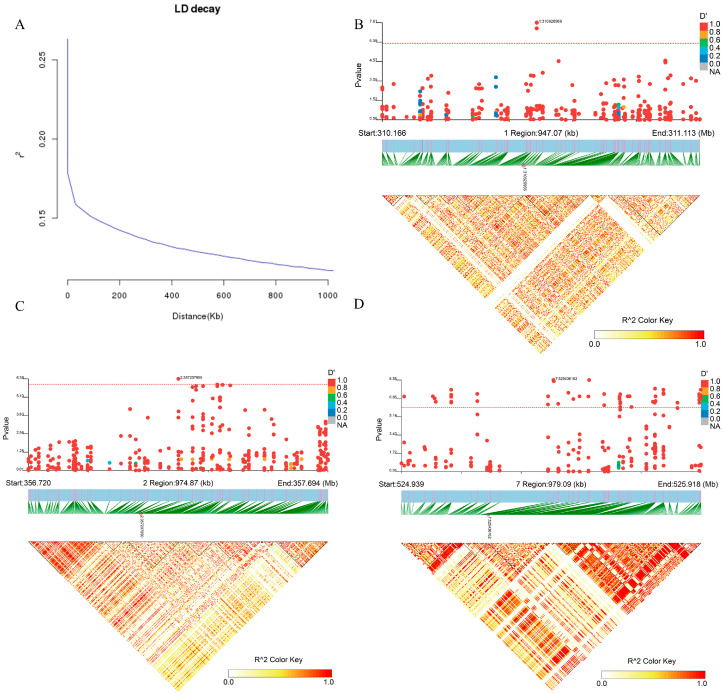
LD decay and candidate regions on chromosomes 1, 2, and 7. (**A**) LD decay analysis; (**B**) GWAS regional association plot with LD heatmap for Chr 1; (**C**) GWAS regional association plot with LD heatmap for Chr 2; (**D**) GWAS regional association plot with LD heatmap for Chr 3. The dashed horizontal line represents the genome-wide significance threshold (*p* < 1.0 × 10^−5^).

Further LD analysis (Figure 6A) showed that the r^2^ value decreased rapidly with increasing physical distance and reached a relatively stable level at approximately 200–300 kb, indicating a high recombination level in the population. Based on LD block analysis (Figure 6B–D), three candidate genomic regions were defined: Chr1 (310.166–311.113 Mb), Chr2 (356.720–357.694 Mb), and Chr7 (524.939–525.918 Mb). Significant SNPs within these regions were clustered and located within strong LD blocks, suggesting that these intervals may contain key genes controlling grain length. Overall, the combined GWAS and LD analyses effectively narrowed down the candidate regions and provided a reliable basis for subsequent candidate gene identification.

### 3.3. Transcriptome Profiling and Differential Expression Analysis

To further elucidate the transcriptional regulatory mechanisms underlying grain length variation and prioritize putative functional candidate genes, transcriptome analysis of grain length was performed at key developmental stages.

#### 3.3.1. Identification of Differentially Expressed Genes

Differential expression analysis was performed between the short-grain genotype WDM720706 (D64) and the long-grain genotype WDM01925 (D180) at four developmental stages (7, 14, 21, and 28 days after flowering), using thresholds of |log_2_FC| ≥ 1 and *p-adj* < 0.05.

**Figure 7:**
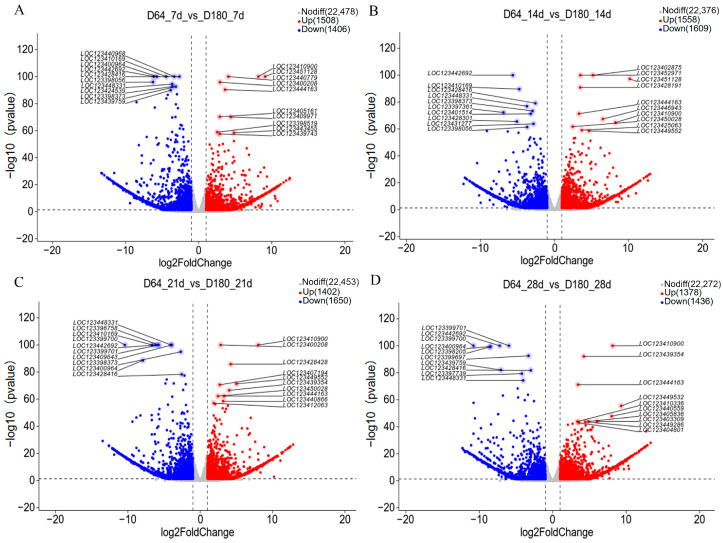
Volcano plots of differentially expressed genes (DEGs) at four grain developmental stages. Each dot represents a gene. Red dots indicate significantly upregulated genes, while blue dots indicate significantly downregulated genes. X-axis: The log_2_ fold change (log_2_FC) of gene expression levels between samples, Y-axis: The −log_10_-transformed adjusted *p*-value (−log_10_ *padj*), reflecting the statistical significance of differential expression. (**A**) Differentially expressed genes (DEGs) between D64 and D180 at 7 days post-treatment (D64_7d_vs_D180_7d); (**B**) DEGs between D64 and D180 at 14 days post-treatment (D64_14d_vs_D180_14d); (**C**) DEGs between D64 and D180 at 21 days post-treatment (D64_21d_vs_D180_21d); (**D**) DEGs between D64 and D180 at 28 days post-treatment (D64_28d_vs_D180_28d).

The results show that a large number of differentially expressed genes (DEGs) were identified at each developmental stage (Figure 7A–D). Specifically, 2914, 3167, 3052, and 2814 DEGs were detected at 7, 14, 21, and 28 days, respectively, including both upregulated and downregulated genes. The numbers of upregulated and downregulated genes were relatively balanced across stages, indicating dynamic transcriptional changes during grain development. This pattern further suggests that grain length is a typical quantitative trait, in which gene expression changes are not dominated by extreme up- or downregulation, but instead occur in a continuous and coordinated manner throughout development.

**Figure 8:**
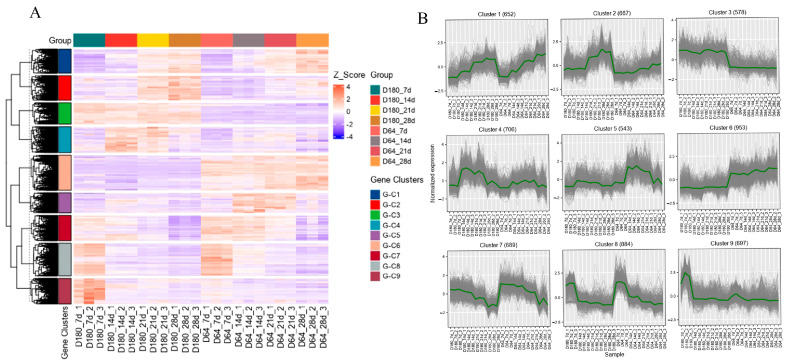
Clustering and expression trends of DEGs. (**A**) Clustering analysis of DEGs; (**B**) Trend Analysis.

Hierarchical clustering and expression trend analysis (Figure 8) showed that gene expression patterns were highly consistent among biological replicates, while clear differences were observed between the two genotypes, indicating good data reliability. Among the identified expression clusters, cluster 8 showed a pronounced stage-specific expression shift, suggesting that this cluster may contain key genes involved in grain length regulation.

#### 3.3.2. Functional Enrichment Analysis of Differentially Expressed Genes Based on GO and KEGG

**Figure 9:**
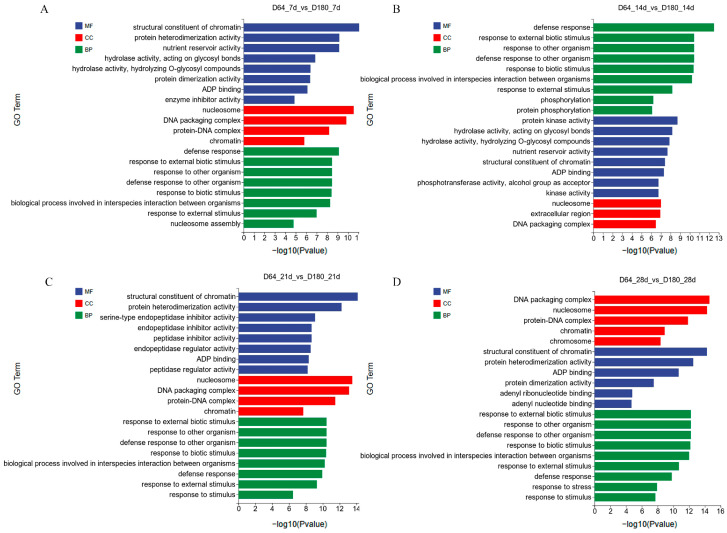
Hierarchical clustering and Gene Ontology (GO) enrichment analysis of differentially expressed genes (DEGs). GO enrichment analysis was performed to classify DEGs into three categories: biological process (BP), cellular component (CC), and molecular function (MF). (**A**) GO enrichment analysis of 7 d; (**B**) GO enrichment analysis of 14 d (**C**) GO enrichment analysis of 21 d (**D**) GO enrichment analysis of 28 d.

GO enrichment analysis (Figure 9A–D) showed that DEGs were significantly enriched in multiple functional categories across different developmental stages. In the molecular function category, DEGs were mainly enriched in structural constituent of chromatin, protein heterodimerization activity and ADP binding. In the cellular component category, DEGs were enriched in nucleosome, DNA packaging complex, and protein–DNA complex, suggesting the importance of chromatin organization.

In the biological process category, DEGs were enriched in defense response and stimulus-related processes, indicating that grain development may be associated with environmental response pathways. In addition, enrichment of protein phosphorylation and kinase activity increased over time, suggesting that signal transduction processes became progressively more active during grain development.

**Figure 10:**
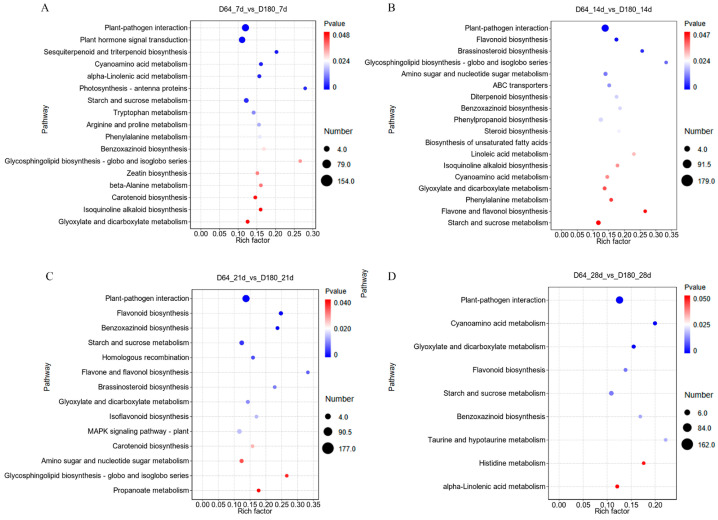
KEGG pathway enrichment analysis. X-axis: Rich factor, defined as the ratio of the number of differentially expressed genes mapped to a given pathway to the total number of genes annotated in that pathway; Y-axis: KEGG pathways, Dot size corresponds to the number of enriched differentially expressed genes (upregulated or downregulated, depending on the input gene set), and color gradient indicates the statistical significance of enrichment. (**A**) KEGG enrichment analysis of 7 d; (**B**) KEGG enrichment analysis of 14 d (**C**) KEGG enrichment analysis of 21 d (**D**) KEGG enrichment analysis of 28 d.

KEGG enrichment analysis across four developmental stages (7, 14, 21, and 28 days after flowering) revealed both conserved and stage-specific pathway dynamics associated with barley grain length formation (Figure 10A–D). Notably, the pathway “Plant–pathogen interaction” was consistently enriched across all stages, representing the most recurrent pathway and suggesting that stress-related signaling is closely integrated with the regulation of grain length development.

Multiple pathways showed elevated gene numbers and rich factors, with the highest enrichment intensity observed at the 14-day stage. Key pathways, including “Flavonoid biosynthesis,” “Brassinosteroid biosynthesis,” and “Starch and sucrose metabolism,” were significantly enriched, indicating that this stage represents a critical regulatory window during which hormone signaling and carbon metabolism coordinately influence cell division and elongation, thereby contributing to grain length determination.

At the 7-day stage, enriched pathways were primarily associated with energy metabolism, such as photosynthesis, reflecting early developmental processes that establish cellular growth potential. The 21-day stage showed increased pathway diversity, including “MAPK signaling pathway,” suggesting enhanced signal integration and regulatory complexity. In contrast, the 28-day stage displayed fewer enriched pathways, mainly related to amino acid and lipid metabolism, indicating a shift toward grain maturation.

Overall, these results suggest that barley grain length is regulated through a temporally coordinated network involving early energy supply, mid-stage hormonal and metabolic control, and late-stage maturation processes. Collectively, these results indicate that grain length formation is regulated by complex metabolic and signaling networks.

#### 3.3.3. Classification and Expression Profiling of Differentially Expressed Transcription Factors

**Figure 11:**
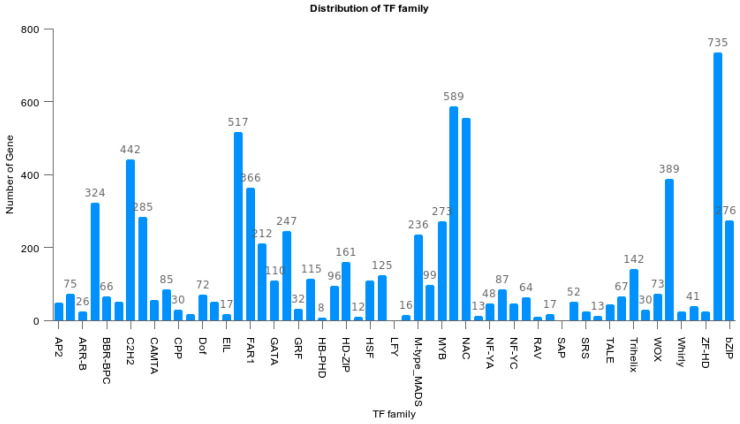
Transcription factor (TF) family distribution.

To further elucidate the relationship between barley grain length phenotypes and transcription factors, we performed classification and expression profiling of differentially expressed transcription factors between the short-grain genotype WDM720706 (D64) and the long-grain genotype WDM01925 (D180). The results showed that different TF families varied greatly in abundance, with bZIP, MYB, and AP2/ERF families being the most represented, suggesting their important roles in transcriptional regulation (Figure 11).

**Figure 12:**
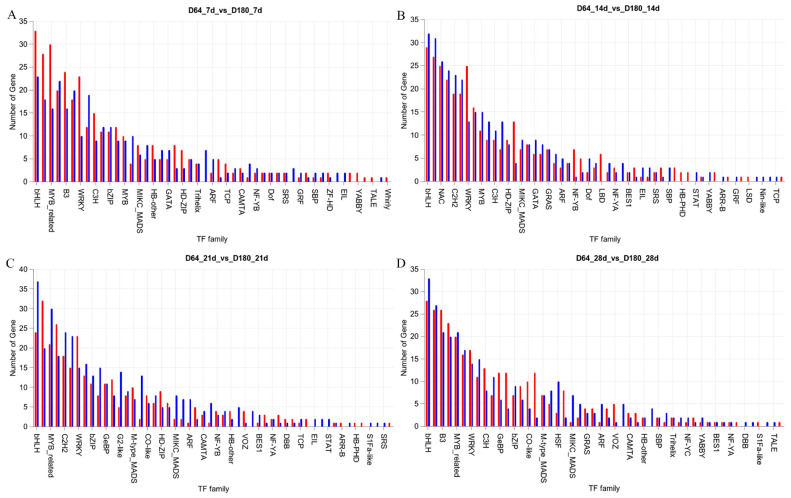
Distribution of differentially expressed transcription factors at different developmental stages. (**A**) Distribution of differentially expressed transcription factors of 7 d; (**B**) Distribution of differentially expressed transcription factors of 14 d (**C**) Distribution of differentially expressed transcription factors of 21 d (**D**) Distribution of differentially expressed transcription factors of 28 d.

Further analysis of different developmental stages (Figure 12A–D) showed that bHLH, MYB, NAC, and WRKY families were consistently enriched, with the highest number of DEGs observed at early stages (7 and 14 days), followed by a gradual decrease.

In addition, both upregulated and downregulated TFs were observed within the same families, suggesting potential bidirectional regulatory roles. Based on their known functions, these TFs may regulate cell division, cell elongation, hormone signaling, and carbon metabolism, thereby contributing to grain development and grain length formation.

### 3.4. Integrated GWAS and RNA-Seq Analysis Identifies Key Candidate Genes

To improve the accuracy of candidate gene identification, GWAS and RNA-seq data were integrated for multi-omics analysis. Based on GWAS results, a total of 84 candidate genes were identified within significant association intervals. Among these, three key candidate genes (*LOC123412467*, *LOC123408579*, and *LOC123407599*) were initially selected based on functional annotation and previous studies.

**Figure 13:**
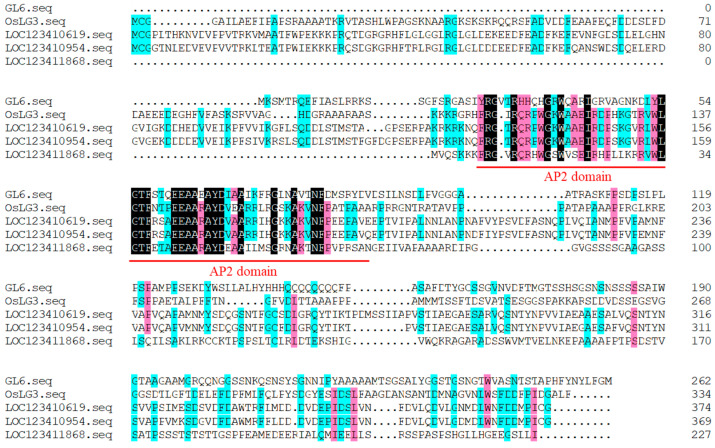
Protein sequence alignment of candidate genes with known grain-length genes. Different colors indicate nucleotide variations. The part underlined in red represents the domain.

To further validate these candidates at the protein level, protein sequences encoded by genes within the candidate regions were extracted and compared with known grain length-related proteins. The results show that *LOC123410619*, *LOC123410954*, and *LOC123411868* exhibited high sequence similarity and contained conserved domains such as AP2 (Figure 13), suggesting their potential roles in transcriptional regulation.

**Figure 14:**
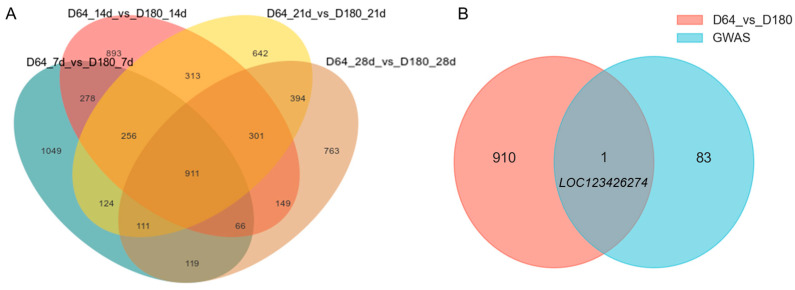
Venn diagram of GWAS candidate genes and DEGs. (**A**) Venn diagram of differentially expressed genes (DEGs) across four developmental stages; (**B**) Venn diagram of GWAS-identified genes and common DEGs across the four developmental stages. In (**A**), different colors represent DEGs identified at 7 d, 14 d, 21 d, and 28 d, respectively. In (**B**), green represents genes identified by GWAS, while red represents common DEGs shared across the four developmental stages.

At the expression level, DEGs identified from RNA-seq were intersected with the 84 GWAS candidate genes. The results show that only one gene, *LOC123426274*, was identified as a common candidate (Figure 14). This gene exhibited consistent downregulation across multiple developmental stages, suggesting a potential negative regulatory role in grain development.

Based on GWAS signals, sequence homology, and expression profiles, a total of seven key candidate genes were finally identified: *LOC123412467*, *LOC123408579*, *LOC123407599*, *LOC123410619*, *LOC123410954*, *LOC123411868*, and *LOC123426274*. These results demonstrate that the integration of multi-omics data effectively improved the accuracy of candidate gene identification.

### 3.5. Sequence Variation Analysis of Candidate Genes

To further validate the functional relevance of candidate genes at the sequence level, targeted sequencing was performed on 60 accessions with extreme grain length phenotypes. The results show that extensive genetic variation, including SNPs and InDels, was present in both coding and regulatory regions of the candidate genes. Among them, *LOC123411868* exhibited a relatively high level of sequence variation (Figure 16A).

**Figure 15:**
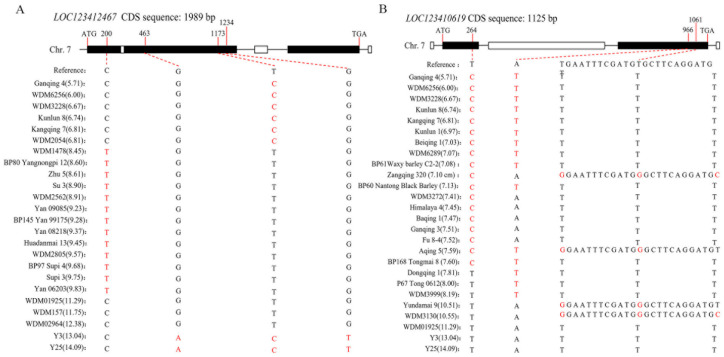
Sequence variation analysis of two candidate genes. (**A**) Sequence comparison of *LOC123412467*; (**B**)Sequence comparison of *LOC123410619*. Red bases indicate differences from the reference sequence, and dashed lines are used solely to indicate the direction of the arrows.

**Figure 16:**
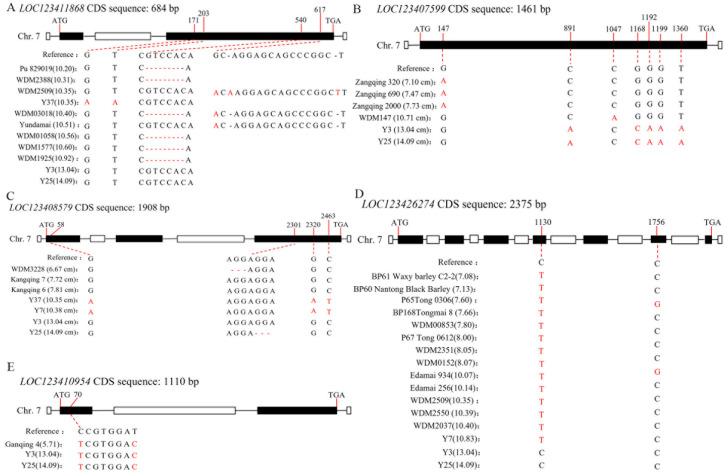
Sequence variation analysis of five candidate genes. (**A**) Sequence comparison of *LOC123411868*; (**B**) Sequence comparison of *LOC123407599*; (**C**) Sequence comparison of *LOC123408579*; (**D**) Sequence comparison of *LOC123426274*; (**E**) Sequence comparison of *LOC123410954.* Red bases indicate differences from the reference sequence, and dashed lines are used solely to indicate the direction of the arrows.

Further analysis indicated that some variants were located in coding regions and resulted in amino acid substitutions (nonsynonymous mutations), which may affect protein structure and function. Notably, *LOC123412467* and *LOC123410619* showed consistent allelic differences between long- and short-grain accessions, suggesting a strong association with grain length variation (Figure 15). In contrast, variation patterns in *LOC123426274*, *LOC123408579*, *LOC123407599*, and *LOC123410954* were less clearly associated with grain length and were partly attributable to differences from wild-type accessions (Figure 16B–E).

Combined with GWAS results, these variants co-localized with significant association signals, further supporting the reliability of the identified candidate genes. Overall, sequence variation analysis provided additional molecular evidence for the functional relevance of key candidate genes.

## 4. Discussion

### 4.1. Genetic Architecture of Barley Grain Length Revealed by GWAS

Grain length in barley is a typical quantitative trait governed by polygenic effects and complex regulatory networks [6,42]. In the present study, genome-wide association analysis identified 84 stable SNP loci significantly associated with grain length across multiple environments, with a pronounced enrichment on chromosome 7. This non-random chromosomal distribution suggests that major-effect loci are embedded within a broader polygenic framework and may function as key regulatory hubs within the genetic network controlling grain size, consistent with previous findings in barley and other cereal crops [1,43,44]. The strong association peak detected on chromosome 7, together with the high density and clustering of significant SNPs, indicates that this region likely represents a regulatory hotspot for grain size determination.

The relatively rapid decay of linkage disequilibrium (LD) observed in this study (approximately 200–300 kb) further supports the high mapping resolution of the population, enabling more precise delimitation of candidate intervals [8,45,46]. Compared with biparental mapping populations, natural populations used in GWAS provide greater allelic diversity and finer resolution, although they are more susceptible to confounding effects arising from population structure [47,48]. The strong concordance between Manhattan and Q–Q plots indicates that the mixed linear model effectively accounted for such confounding factors, including population structure and kinship [46].

Taken together, these results demonstrate that the barley natural population used in this study provides sufficient genetic diversity and mapping resolution for reliable GWAS analysis, and that the identified loci, particularly those enriched on chromosome 7, represent robust and biologically meaningful targets for subsequent candidate gene identification and functional validation [9,10,49].

### 4.2. Dynamic Transcriptomic Reprogramming During Grain Development

RNA-seq analysis across four key developmental stages revealed extensive transcriptional divergence between long- and short-grain genotypes, consistent with previous findings in cereal crops [16,18]. Thousands of differentially expressed genes (DEGs) were identified at each stage, with relatively balanced proportions of up- and downregulated genes. This pattern indicates coordinated and dynamic transcriptional regulation rather than unidirectional shifts, which is characteristic of complex quantitative traits [50].

Functional enrichment analyses showed that DEGs were significantly associated with chromatin organization, transcriptional regulation, and diverse metabolic processes. The enrichment of terms such as “structural constituent of chromatin” and “nucleosome” highlights the potential role of epigenetic regulation in grain development. Chromatin remodeling has been demonstrated to influence transcriptional accessibility and gene expression dynamics during seed development, thereby affecting cell proliferation and expansion [51,52,53].

Furthermore, KEGG pathway analysis indicated significant enrichment in plant hormone signal transduction, MAPK signaling pathway, and starch and sucrose metabolism. These pathways are known to coordinate key developmental processes, including cell division, cell elongation, and assimilate allocation, which collectively determine grain size [24,26]. The consistent enrichment of “plant–pathogen interaction” pathways suggests potential cross-talk between developmental and stress-response signaling networks, reflecting the integration of growth regulation and environmental adaptability during grain development [54,55].

### 4.3. Transcription Factor Networks Underlying Grain Length Regulation

Transcription factors (TFs) are central regulators of gene expression and play pivotal roles in plant developmental processes [56]. In this study, several TF families, including bZIP, MYB, and AP2/ERF, were prominently enriched among the DEGs, consistent with their established roles in regulating grain size and developmental pathways [57].

Temporal expression profiling revealed that TF activity was most pronounced during early grain development, particularly at 7 and 14 days after flowering. This observation supports the hypothesis that early developmental stages represent a critical window during which transcriptional regulation exerts a decisive influence on final grain size [58,59]. The subsequent decline in TF activity at later stages likely reflects a developmental transition from active cell proliferation to grain filling and maturation.

Importantly, TFs rarely function in isolation; instead, they operate within complex regulatory networks that integrate hormonal signals and environmental cues [56,60]. The coexistence of both upregulated and downregulated TFs within the same families suggests the presence of feedback regulation and functional diversification, contributing to the fine-tuning of gene expression during grain development. Notably, AP2/ERF family members, which have been widely implicated in grain size regulation in rice and wheat (e.g., *GL7/GW7*), were also enriched in this study, indicating conserved regulatory mechanisms across cereal species [21,27].

### 4.4. Integrated Multi-Omics Analysis Identifies High-Confidence Candidate Genes

To overcome the inherent limitations of single-omics approaches, this study integrated GWAS and RNA-seq data to refine candidate gene selection [22]. Several candidate genes were prioritized based on their genomic positions, functional annotations, and homology to known grain size regulators.

Interestingly, only one gene overlapped between GWAS-identified candidates and DEGs. This limited overlap is consistent with previous studies indicating that genetic associations do not always directly correspond to transcriptional variation [61]. Such discrepancies may arise because causal variants can influence protein function, regulatory elements, or post-transcriptional processes without necessarily altering gene expression levels.

The integration of positional, functional, and expression data significantly enhances the reliability of candidate gene prioritization and reduces false positives, a major challenge in GWAS-based studies [9]. This multi-layered approach provides a more comprehensive understanding of the genetic basis of complex traits.

### 4.5. Sequence Variation Provides Functional Evidence for Key Candidate Genes

Targeted resequencing of extreme phenotypes revealed abundant SNP and InDel variations within candidate gene regions, providing direct evidence linking genetic variation to phenotypic diversity [28]. Notably, several non-synonymous mutations were identified, which may alter protein structure and function, thereby influencing grain development [62].

The consistent allelic differences observed between long- and short-grain genotypes strongly support the functional relevance of key candidate genes. Similar mechanisms have been reported in rice and wheat, where coding sequence variations in genes such as *GS3* and *GS5* directly affect grain size [24,26]. The co-localization of sequence variants with GWAS signals further strengthens the evidence for their involvement in grain length regulation.

### 4.6. Proposed Regulatory Model and Implications for Molecular Breeding

Based on integrative analyses, a putative regulatory framework for barley grain length can be proposed. In this model, genetic variation at key loci influences transcription factor activity and chromatin structure, which in turn regulate downstream gene expression networks. These networks integrate hormonal signaling pathways, metabolic processes, and environmental responses to control cell proliferation and elongation during early grain development, ultimately determining grain size [44,58].

This proposed model is consistent with conserved mechanisms identified in other cereal crops, such as rice and wheat, where genes like *GS3, GL7*, and *GS5* regulate grain size through transcriptional and hormonal pathways [24,26,27]. The identification of homologous or functionally analogous genes in barley suggests that core regulatory modules governing grain development are evolutionarily conserved.

From a breeding perspective, the candidate genes identified in this study represent valuable targets for marker-assisted selection and genomic selection strategies [63]. In particular, loci with stable effects across environments are of high practical significance for improving yield-related traits. Moreover, the integration of multi-omics data provides an efficient framework for accelerating gene discovery and enhancing breeding efficiency.

### 4.7. Limitations and Future Perspectives

Despite the robustness of the integrative approach, several limitations should be acknowledged. The sample size, although adequate for initial discovery, may limit the detection of rare variants. In addition, RNA-seq analysis was conducted on only two extreme genotypes, which may not fully capture the diversity of transcriptional responses. Furthermore, functional validation of candidate genes remains to be performed.

Future studies should focus on expanding population size, incorporating additional omics layers such as epigenomics and metabolomics, and applying functional validation approaches, including CRISPR/Cas-mediated gene editing [64]. These efforts will further elucidate the molecular mechanisms underlying grain length variation and facilitate the translation of genomic findings into practical applications for crop improvement.

## 5. Conclusions

In this study, an integrated multi-omics approach combining genome-wide association study (GWAS), transcriptome sequencing, protein homology analysis, and targeted resequencing was applied to systematically elucidate the genetic basis of barley grain length. A total of 84 stable SNP loci significantly associated with grain length were identified, with major association signals predominantly distributed on chromosome 7. By integrating genomic localization, differential expression profiling, protein structural homology, and sequence variation analyses, seven high-confidence candidate genes were ultimately identified, including *LOC123412467*, *LOC123408579*, *LOC123407599*, *LOC123410619*, *LOC123410954*, *LOC123411868*, and *LOC123426274*. Notably, *LOC123412467* and *LOC123410619* exhibited consistent allelic variation between long- and short-grain accessions, strongly supporting their potential roles as key regulators of grain length formation.

The major novelty of this study lies in the integration of GWAS, RNA-seq, protein homology comparison, and sequence-level validation to improve the precision and reliability of candidate gene identification for a complex quantitative trait in barley. Furthermore, our results suggest that transcriptional regulation, chromatin remodeling, and hormone-mediated signaling pathways coordinately contribute to early grain development and final grain size determination. These findings provide new insights into the molecular regulatory mechanisms underlying barley grain length and offer valuable genetic resources and candidate targets for future functional validation and molecular breeding aimed at improving yield-related traits in barley.

## Figures and Tables

**Table 1:** Previously cloned genes associated with grain length regulation.

Gene Name	Crop	Chromosome	Function	Domain	References
*GS2/GL2*	Rice	2	Growth-regulating factor OsGRF4	None	[25]
*OsLG3*	Rice	3	AP2/ERF family transcription factor	AP2	[31]
*OsLG3b*	Rice	3	MADS-box transcription factor	None	[32]
*GS3*	Rice	3	transmembrane protein	None	[24]
*qGL3/GL3.1*	Rice	3	protein phosphatase OsPPKL1	None	[33]
*qTGW3/TGW3/GL3.3*	Rice	3	protein kinase OsGsK5/OsSK41	None	[34]
*GL4*	Rice	4	premature termination codon	None	[35]
*GL6*	Rice	6	AP2/ERF family transcription factor	AP2	[36]
*GLW7*	Rice	7	SPL family transcription factor OsSPL13	None	[37]
*GL7/GW7*	Rice	7	TON1-recruit motif protein	None	[27]
*GS9*	Rice	9	a RING-type E3 ubiquitin ligase	None	[38]
*TaGS2*	Wheat		Growth-regulating factor family	None	[39]
*TaGS5-3D*	Wheat		encodes a serine carboxypeptidase	None	[40]
*TaGL3-5A*	Wheat		encodes a protein kinase containing Kelch-like repeat domains	None	[41]

**Table 2:** Phenotypic variation in grain length among 198 barley accessions.

Trail	Mean	SD	CV	Range	Skewness	Kurtosis
2021	8.775	1.088	12.396	5.996~12.635	0.21	−0.11
2022	9.204	1.145	12.439	5.995~12.437	0.42	−0.20

**Table 3:** Information on significant genomic regions identified by GWAS.

Chromosome	Gene ID	Gene-Start	Gene-End
Chr1	*LOC123432985*	310237761	310239981
Chr1	*LOC123433001*	310735492	310746728
Chr1	*LOC123433017*	310736657	310748499
Chr1	*LOC123433033*	310967696	310972052
Chr2	*LOC123426272*	356912989	356916360
Chr2	*LOC123426273*	356984236	356989532
Chr2	*LOC123426275*	356992826	356993314
Chr2	*LOC123426276*	357412255	357415562
Chr2	*LOC123426278*	357421211	357422228
Chr2	*LOC123426274*	356990642	356993016
Chr2	*LOC123426277*	357415721	357416969
Chr7	*LOC123408579*	177647331	177651304
Chr7	*LOC123408821*	178064514	178065889
Chr7	*LOC123412572*	522759136	522762741
Chr7	*LOC123412467*	522815292	522818067
Chr7	*LOC123407599*	522970429	522972262
Chr7	*LOC123410937*	524660929	524664494
Chr7	*LOC123411293*	524704641	524705048
Chr7	*LOC123407999*	524872206	524876166
Chr7	*LOC123411273*	525220669	525221884
Chr7	*LOC123407759*	525236426	525237662
Chr7	*LOC123409210*	525358623	525359295
Chr7	*LOC123413432*	525390798	525395765
Chr7	*LOC123411868*	525633735	525635145
Chr7	*LOC123411869*	525634551	525647654
Chr7	*LOC123413296*	525682470	525684200
Chr7	*LOC123407044*	525872459	525876025
Chr7	*LOC123408506*	526209290	526210442
Chr7	*LOC123412363*	526313607	526314670
Chr7	*LOC123411192*	526829934	526832688
Chr7	*LOC123411191*	526832963	526839590
Chr7	*LOC123407532*	526846433	526850091
Chr7	*LOC123407926*	527102664	527103885
Chr7	*LOC123409214*	528109364	528111923
Chr7	*LOC123409875*	528240247	528247723
Chr7	*LOC123406937*	528636370	528637719
Chr7	*LOC123406935*	528637871	528642185
Chr7	*LOC123408721*	529083763	529087191
Chr7	*LOC123407169*	529090011	529094634
Chr7	*LOC123409023*	529318149	529323827
Chr7	*LOC123407580*	529477214	529478914
Chr7	*LOC123412548*	529481932	529484651
Chr7	*LOC123407431*	529699922	529704589
Chr7	*LOC123409217*	529848031	529851895
Chr7	*LOC123409545*	530093282	530096808
Chr7	*LOC123410619*	530320378	530323638
Chr7	*LOC123412189*	530367406	530368464
Chr7	*LOC123412138*	530525301	530528035
Chr7	*LOC123408669*	530913566	530916443
Chr7	*LOC123411308*	531165488	531169026
Chr7	*LOC123411434*	531241991	531245201
Chr7	*LOC123412125*	531260681	531262795
Chr7	*LOC123410296*	531287876	531289938
Chr7	*LOC123411089*	531440566	531443392
Chr7	*LOC123411961*	531484566	531485340
Chr7	*LOC123409221*	531572372	531574589
Chr7	*LOC123411774*	531586367	531587531
Chr7	*LOC123414426*	531589364	531589457
Chr7	*LOC123414481*	531589715	531589810
Chr7	*LOC123414348*	531590174	531590297
Chr7	*LOC123410859*	531590716	531591532
Chr7	*LOC123414410*	531602509	531602602
Chr7	*LOC123414499*	531602854	531602949
Chr7	*LOC123407029*	531603729	531610010
Chr7	*LOC123407205*	531705784	531709226
Chr7	*LOC123407204*	531754683	531758324
Chr7	*LOC123407033*	524728158	524783776
Chr7	*LOC123412162*	525090142	525092718
Chr7	*LOC123413373*	525349698	525354696
Chr7	*LOC123410938*	525399692	525404025
Chr7	*LOC123411870*	525625003	525634154
Chr7	*LOC123407362*	526167392	526173185
Chr7	*LOC123408874*	526744329	526745843
Chr7	*LOC123410954*	527104822	527107791
Chr7	*LOC123407021*	527907338	527910583
Chr7	*LOC123408720*	529067322	529079110
Chr7	*LOC123409025*	529316054	529318098
Chr7	*LOC123413096*	529705091	529712329
Chr7	*LOC123412562*	529889629	529894039
Chr7	*LOC123412826*	530087217	530088196
Chr7	*LOC123407612*	530217810	530219790
Chr7	*LOC123412635*	530787796	530797189
Chr7	*LOC123407123*	531471540	531481807
Chr7	*LOC123409906*	531575016	531580218

## Data Availability

Data are contained within the article.

## Abbreviations

The following abbreviations are used in this manuscript:

GWAS	Genome-wide association studies
RNA-seq	RNA sequencing
LD	linkage disequilibrium
DEGs	Differentially expressed genes

## Appendix A

**Table A1:** Primers used for candidate gene localization in this study.

Gene Name	Name	Forward Primer (5′-3′)	Reverse Primer (5′-3′)
*LOC123412467*	CA-1	GCTGCCACTATCCCAGTAG	CGGACAGGTTGTTGTAAGAG
	CA-2	TTCCTGCTGGTTCTTTCC	CCCTCCCTGTAATAAATG
	CA-3	TGGGTGCCAACTTACTAC	AGGTGGTGTTCAAATGTG
*LOC123407599*	CB-1	AAGACTTCCTATTTAGTTCGGCTG	CTTGGCAATCCTTGGTGC
*LOC123408579*	UP-1	GCCTTCTTCTGTTCTGTC	ACCCACCGATACTATGTG
	UP-2	TCCTCATCCTCATCCGTG	ACCCACCGATACTATGTG
	UP-3	CTCCTTTAGCCGTTTCAC	AACCACACAGAGATAGATAGG
	UP-4	GTGAAGGAGACGGAAGTG	GTTCACAAGATAGAAATGCC
*LOC123426274*	4-1	ATACCTCCTACCTTTCGTC	CCTATCAGTTCATCAGGC
	4-2	ATTTCAGCACAGGCATTG	GTGGTAGGAGATTGAGCG
*LOC123410954*	5-1	CATCCCACTTCTCCTAAG	CCTTTGTCCTGGTAACTAAC
	5-2	ATGACCCATCAGATTCGG	GCGACGACATCAAAGTTG
*LOC123410619*	6-1	CCCATTCATTCATTACCG	TCTACTACCTCCGTCCCAG
	6-2	CTTGACCCATCAGGTTCG	TGGATTACAAAGGAAACTCAGG
*LOC123411868*	7-a	TCACTCACTCACTCACTCG	AATGGAGAGATACTCGGC

## References

[1] Mascher M., Gundlach H., Himmelbach A., Beier S., Twardziok S.O., Wicker T., Radchuk V., Dockter C., Hedley P.E., Russell J. (2017). A chromosome conformation capture ordered sequence of the barley genome. Nature.

[2] Pourkheirandish M., Komatsuda T. (2007). The importance of barley genetics and domestication in a global perspective. Ann. Bot..

[3] Zhou Y., Lu D.F., Li C.Y., Luo J.H., Zhu B.F., Zhu J.J., Shangguan Y.Y., Wang Z.X., Sang T., Zhou B. (2012). Genetic control of seed shattering in rice by the APETALA2 transcription factor SHATTERING ABORTION1. Plant Cell.

[4] Li H., Wang Y.P., Qiao W.H., Zhu Z., Wang Z.Y., Tian Y.L., Liu S.J., Wan J.M., Liu L.L. (2024). Identification of a novel locus qGW12/OsPUB23 regulating grain shape and weight in rice (*Oryza sativa* L.). Theor. Appl. Genet..

[5] Zhao D.S., Zhang C.G., Li Q.F., Liu Q.Q. (2022). Genetic control of grain appearance quality in rice. Biotechnol. Adv..

[6] Huang X.H., Wei X.H., Sang T., Zhao Q., Feng Q., Zhao Y., Li C.Y., Zhu C.R., Lu T.T., Zhang Z.W. (2010). Genome-wide association studies of 14 agronomic traits in rice landraces. Nat. Genet..

[7] Mackay T.F.C., Stone E.A., Ayroles J.F. (2009). The genetics of quantitative traits: Challenges and prospects. Nat. Rev. Genet..

[8] Flint-Garcia S.A., Thornsberry J.M., Buckler E.S. (2003). Structure of linkage disequilibrium in plants. Annu. Rev. Plant Biol..

[9] Tam V., Patel N., Turcotte M., Bossé Y., Paré G., Meyre D. (2019). Benefits and limitations of genome-wide association studies. Nat. Rev. Genet..

[10] Visscher P.M., Wray N.R., Zhang Q., Sklar P., McCarthy M.I., Brown M.A., Yang J. (2017). 10 years of GWAS discovery: Biology, function, and translation. Am. J. Hum. Genet..

[11] Hu X., Zuo J.F., Wang J.B., Liu L.P., Sun G.L., Li C.D., Ren X.F., Sun D.F. (2018). Multi-locus genome-wide association studies for agronomic traits in barley. Front. Plant Sci..

[12] Nice L.M., Steffenson B.J., Brown-Guedira G.L., Akhunov E.D., Liu C., Kono T.J.Y., Morrell P.L., Blake T.K., Horsley R.D., Smith K.P. (2016). Development and genetic characterization of an advanced backcross-nested association mapping population of wild × cultivated barley. Genetics.

[13] Hong Y., Zhang M.G., Zhu J., Zhang Y.H., Lv C., Guo B.J., Wang F.F., Xu R.G. (2024). Genome-wide association studies reveal novel loci for grain size in two-rowed barley. Theor. Appl. Genet..

[14] Bayer M.M., Rapazote-Flores P., Ganal M., Hedley P.E., Macaulay M., Plieske J., Ramsay L., Russell J., Shaw P.D., Thomas W.T.B. (2017). Development and evaluation of a barley 50k iSelect SNP array. Front. Plant Sci..

[15] Nordborg M., Weigel D. (2008). Next-generation genetics in plants. Nature.

[16] Wang Z., Gerstein M., Snyder M. (2009). RNA-Seq: A revolutionary tool for transcriptomics. Nat. Rev. Genet..

[17] Mortazavi A., Williams B.A., McCue K., Schaeffer L., Wold B. (2008). Mapping and quantifying mammalian transcriptomes by RNA-Seq. Nat. Methods.

[18] Li N., Xu R., Li Y.H. (2019). Molecular networks of seed size control. Annu. Rev. Plant Biol..

[19] Rangan P., Furtado A., Henry R. (2020). Transcriptome profiling of wheat genotypes under heat stress during grain filling. J. Cereal Sci..

[20] Evans P., Nagai T., Konkashbaev A., Zhou D., Knapik E.W., Gamazon E.R. (2024). Transcriptome-wide association studies (TWAS): Methodologies, applications, and challenges. Curr. Protoc..

[21] Wu X., Li Y.X., Shi Y.S., Song Y.C., Zhang D.F., Li C.H., Buckler E.S., Li Y., Zhang Z.W., Wang T.Y. (2016). Joint-linkage mapping and GWAS reveal genetic loci regulating maize inflorescence size. Plant Biotechnol. J..

[22] Kremling K.A.G., Chen S.-Y., Su M.-H., Lepak N.K., Romay M.C., Swarts K.L., Lu F., Lorant A., Bradbury P.J., Buckler E.S. (2018). Dysregulation of expression correlates with rare-allele burden and fitness loss in maize. Nat. Genet..

[23] Luan H.Y., Gao J.J., Wu Y.H., Yang J.H., Shen Y., Sun M.L., Liu F.F., Xu M., Xu X., Sun M. (2025). Identification of candidate genes for grain size in barley through combined GWAS and transcriptome analysis. J. Plant Res..

[24] Fan C.C., Xing Y.Z., Mao H.L., Lu T.T., Han B., Xu C.G., Li X.H., Zhang Q.F. (2006). GS3, a major QTL for grain length and weight in rice. Theor. Appl. Genet..

[25] Hu J., Wang Y.X., Fang Y.X., Zeng L.J., Xu J., Yu H.P., Shi Z.Y., Pan J.J., Zhang D., Kang S.J. (2015). A rare allele of GS2 enhances grain size and yield in rice. Mol. Plant.

[26] Li Y.B., Fan C.C., Xing Y.Z., Jiang Y.H., Luo L.J., Sun L., Shao D., Xu C.J., Li X.H., Xiao J.H. (2011). Natural variation in GS5 regulates grain size and yield. Nat. Genet..

[27] Wang Y.X., Xiong G.S., Hu J., Jiang L., Yu H., Xu J., Fang Y.X., Zeng L.J., Xu E.B., Xu J. (2015). Copy number variation at GL7 locus contributes to grain size diversity. Nat. Genet..

[28] Alonso-Blanco C., Aarts M.G.M., Bentsink L., Keurentjes J.J.B., Reymond M., Vreugdenhil D., Koornneef M. (2009). What Has Natural Variation Taught Us about Plant Development, Physiology, and Adaptation?. Plant Cell.

[29] Rockman M.V. (2012). The QTN Program and the Alleles That Matter for Evolution: All That’s Gold Does Not Glitter. Evolution.

[30] Hasin Y., Seldin M., Lusis A. (2017). Multi-omics approaches to disease. Genome Biol..

[31] Yu J.P., Xiong H.Y., Zhu X.Y., Zhang H.L., Li H.H., Miao J.L., Wang W.S., Tang Z.S., Zhang Z.Y., Yao G.X. (2017). OsLG3 contributing to rice grain length and yield was mined by Ho-LAMap. BMC Biol..

[32] Yu J.P., Miao J.L., Zhang Z.Y., Xiong H.Y., Zhu X.Y., Sun X.M., Pan Y.H., Liang Y.T., Zhang Q., Abdul R.M. (2018). Alternative splicing of OsLG3b controls grain length and yield in japonica rice. Plant Biotechnol. J..

[33] Qi P., Lin Y.S., Song X.J., Shen J.B., Huang W., Shan J.X., Zhu M.Z., Jiang L.W., Gao P.J., Lin H.X. (2012). The novel quantitative trait locus GL3.1 controls rice grain size and yield by regulating Cyclin-T1;3. Cell Res..

[34] Hu Z.J., Lu S.J., Wang M.J., He H.H., Sun L., Wang H.R., Liu X.H., Jiang L., Sun J.L., Xin X.Y. (2018). A novel QTL qTGW3 encodes the GSK3/SHAGGY-like kinase OsGSK5/OsSK41 that interacts with OsARF4 to negatively regulate grain size and weight in rice. Mol. Plant.

[35] Wu W.G., Liu X.Y., Wang M.H., Meyer R.S., Luo X.J., Ndjiondjop M.N., Tan L.B., Zhang J.W., Wu J.Z., Cai H.W. (2017). A single-nucleotide polymorphism causes smaller grain size and loss of seed shattering during African rice domestication. Nat. Plants.

[36] Wang T., Zou T., He Z.Y., Yuan G.Q., Luo T., Zhu J., Liang Y.Y., Deng Q.M., Wang S.Q., Zheng A.P. (2019). Grain length and awn 1 negatively regulates grain size in rice. J. Integr. Plant Biol..

[37] Si L.Z., Chen J.Y., Huang X.H., Gong H., Luo J.H., Hou Q.Q., Zhou T.Y., Lu T.T., Zhu J.J., Shangguan Y.Y. (2016). OsSPL13 controls grain size in cultivated rice. Nat. Genet..

[38] Zhao D.S., Li Q.F., Zhang C.Q., Zhang C., Yang Q.Q., Pan L.X., Ren X.Y., Lu J., Gu M.H., Liu Q.Q. (2018). GS9 acts as a transcriptional activator to regulate rice grain shape and appearance quality. Nat. Commun..

[39] Hu M.Y., Zhao X.Q., Liu Q., Hong X., Zhang W., Zhang Y.J., Sun L.J., Li H., Tong Y.P. (2018). Transgenic expression of plastidic glutamine synthetase increases nitrogen uptake and yield in wheat. Plant Biotechnol. J..

[40] Zhang Y.Y., Miao H.X., Xiao Y., Wang C., Zhang J.J., Shi X.X., Xie S.F., Wang C.Y., Li T.D., Deng P.C. (2023). An intron-located single nucleotide variation of TaGS5-3D is related to wheat grain size through accumulating intron retention transcripts. Theor. Appl. Genet..

[41] Yang J., Zhou Y.J., Wu Q.H., Chen Y.X., Zhang P.P., Zhang Y.E., Hu W.G., Wang X.C., Zhao H., Dong L.L. (2019). Molecular characterization of a novel TaGL3-5A allele and its association with grain length in wheat (*Triticum aestivum* L.). Theor. Appl. Genet..

[42] Watt C., Zhou G., McFawn L.A., Li C. (2020). Fine mapping qGL2H, a major locus controlling grain length in barley (Hordeum vulgare L.). Theor. Appl. Genet..

[43] Xu X., Sharma R., Tondelli A., Russell J., Comadran J., Schnaithmann F., Pillen K., Kilian B., Cattivelli L., Thomas W.T.B. (2018). Genome-wide association analysis of grain yield-associated traits in a pan-European barley cultivar collection. Plant Genome.

[44] Li N., Li Y.H. (2016). Signaling pathways of seed size control in plants. Curr. Opin. Plant Biol..

[45] Korte A., Farlow A. (2013). The advantages and limitations of trait analysis with GWAS: A review. Plant Methods.

[46] Yu J.M., Pressoir G., Briggs W.H., Vroh Bi I., Yamasaki M., Doebley J.F., McMullen M.D., Gaut B.S., Nielsen D.M., Holland J.B. (2006). A unified mixed-model method for association mapping that accounts for multiple levels of relatedness. Nat. Genet..

[47] Pasam R.K., Sharma R., Malosetti M., van Eeuwijk F.A., Haseneyer G., Kilian B., Graner A. (2012). Genome-wide association studies for agronomical traits in a worldwide spring barley collection. BMC Plant Biol..

[48] Price A.L., Patterson N.J., Plenge R.M., Weinblatt M.E., Shadick N.A., Reich D. (2006). Principal components analysis corrects for stratification in genome-wide association studies. Nat. Genet..

[49] Brachi B., Morris G.P., Borevitz J.O. (2011). Genome-wide association studies in plants: The missing heritability is in the field. Genome Biol..

[50] Zhang X.J., Wang J.F., Huang J., Lan H.X., Wang C.L., Yin C.F., Wu Y.Y., Tang H.J., Qian Q., Li J.F. (2012). Rare allele of OsPPKL1 associated with grain length causes extra-large grain and a significant yield increase in rice. Proc. Natl. Acad. Sci. USA.

[51] Li G., Liu S., Wang J., He J., Huang H., Zhang Y., Xu L. (2014). ISWI proteins participate in the genome-wide nucleosome distribution in Arabidopsis. Plant J..

[52] Zhao L., Kim Y.J., Dinh T.T., Chen X.M. (2007). miR172 regulates stem cell fate and defines the inner boundary of APETALA3 and PISTILLATA expression domain in Arabidopsis floral meristems. Plant J..

[53] Han S.K., Wagner D. (2014). Role of chromatin in water stress responses in plants. J. Exp. Bot..

[54] Huot B., Yao J., Montgomery B.L., He S.Y. (2014). Growth–defense tradeoffs in plants: A balancing act to optimize fitness. Mol. Plant..

[55] Li Y.J., Huang K., Zhang L.J., Zhang B.L., Duan P.G., Zhang G.Z., Huang X.H., Zhou C., Han N.N., Zheng L.Y. (2025). A molecular framework for the GS2-SUG1 module-mediated control of grain size and weight in rice. Nat. Commun..

[56] Singh K.B., Foley R.C., Oñate-Sánchez L. (2002). Transcription factors in plant defense and stress responses. Curr. Opin. Plant Biol..

[57] Guo T., Chen K., Dong N.Q., Shi C.L., Ye W.W., Gao J.P., Shan J.X., Lin H.X. (2018). Grain size and number1 negatively regulates the OsMKKK10–OsMKK4–OsMPK6 cascade to coordinate the trade-off between grain number per panicle and grain size in rice. Plant Cell.

[58] Sabelli P.A., Larkins B.A. (2009). The development of endosperm in grasses. Plant Physiol..

[59] Olsen O.A. (2004). Nuclear endosperm development in cereals and Arabidopsis thaliana. Plant Cell.

[60] Verma V., Ravindran P., Kumar P.P. (2016). Plant hormone-mediated regulation of stress responses. BMC Plant Biol..

[61] Albert F.W., Kruglyak L. (2015). The role of regulatory variation in complex traits and disease. Nat. Rev. Genet..

[62] Sun X.W., Liu D.Y., Zhang X.F., Li W.B., Liu H., Hong W.G., Jiang C.B., Guan N., Ma C.X., Zeng H.P. (2013). SLAF-seq: An efficient method of large-scale de novo SNP discovery and genotyping using high-throughput sequencing. PLoS ONE.

[63] Alemu A., Åstrand J., Montesinos-López O.A., Isidro y Sánchez J., Fernández-Gónzalez J., Tadesse W., Vetukuri R.R., Carlsson A.S., Ceplitis A., Crossa J. (2017). Genomic selection in plant breeding: Key factors shaping two decades of progress. Mol. Plant.

[64] Zeb A., Sohail A., Tang S.J., Sheng Z.H., Hu P.S. (2025). CRISPR/Cas9-mediated mutations of GS3 and GW5 positively regulate grain size and grain width in rice (*Oryza sativa* indica). Ecol. Genet. Genom..

